# Stabilization Effect of Intrinsically Disordered Regions on Multidomain Proteins: The Case of the Methyl-CpG Protein 2, MeCP2

**DOI:** 10.3390/biom11081216

**Published:** 2021-08-16

**Authors:** David Ortega-Alarcon, Rafael Claveria-Gimeno, Sonia Vega, Olga C. Jorge-Torres, Manel Esteller, Olga Abian, Adrian Velazquez-Campoy

**Affiliations:** 1 Institute of Biocomputation and Physics of Complex Systems (BIFI), Joint Units IQFR-CSIC-BIFI, and GBsC-CSIC-BIFI, Universidad de Zaragoza, 50018 Zaragoza, Spain; dortega@bifi.es (D.O.-A.); rafacg@certest.es (R.C.-G.); svega@bifi.es (S.V.); 2 Instituto Aragonés de Ciencias de la Salud (IACS), 50009 Zaragoza, Spain; 3 Instituto de Investigación Sanitaria de Aragón (IIS Aragón), 50009 Zaragoza, Spain; 4 Josep Carreras Leukaemia Research Institute (IJC), 08916 Badalona, Spain; ojorge@carrerasresearch.org (O.C.J.-T.); mesteller@carrerasresearch.org (M.E.); 5 Centro de Investigacion Biomedica en Red Cancer (CIBERONC), 28029 Madrid, Spain; 6 Institucio Catalana de Recerca i Estudis Avançats (ICREA), 08010 Barcelona, Spain; 7 Physiological Sciences Department, School of Medicine and Health Sciences, University of Barcelona (UB), l’Hospitalet de Llobregat, 08907 Barcelona, Spain; 8 Centro de Investigación Biomédica en Red en el Área Temática de Enfermedades Hepáticas y Digestivas (CIBERehd), 28029 Madrid, Spain; 9 Departamento de Bioquímica y Biología Molecular y Celular, Universidad de Zaragoza, 50009 Zaragoza, Spain; 10 Fundación ARAID, Gobierno de Aragón, 50009 Zaragoza, Spain

**Keywords:** MeCP2, intrinsically disordered proteins, structural stability, thermal and chemical denaturation, differential scanning calorimetry

## Abstract

Intrinsic disorder plays an important functional role in proteins. Disordered regions are linked to posttranslational modifications, conformational switching, extra/intracellular trafficking, and allosteric control, among other phenomena. Disorder provides proteins with enhanced plasticity, resulting in a dynamic protein conformational/functional landscape, with well-structured and disordered regions displaying reciprocal, interdependent features. Although lacking well-defined conformation, disordered regions may affect the intrinsic stability and functional properties of ordered regions. MeCP2, methyl-CpG binding protein 2, is a multifunctional transcriptional regulator associated with neuronal development and maturation. MeCP2 multidomain structure makes it a prototype for multidomain, multifunctional, intrinsically disordered proteins (IDP). The methyl-binding domain (MBD) is one of the key domains in MeCP2, responsible for DNA recognition. It has been reported previously that the two disordered domains flanking MBD, the N-terminal domain (NTD) and the intervening domain (ID), increase the intrinsic stability of MBD against thermal denaturation. In order to prove unequivocally this stabilization effect, ruling out any artifactual result from monitoring the unfolding MBD with a local fluorescence probe (the single tryptophan in MBD) or from driving the protein unfolding by temperature, we have studied the MBD stability by differential scanning calorimetry (reporting on the global unfolding process) and chemical denaturation (altering intramolecular interactions by a different mechanism compared to thermal denaturation).

## 1. Introduction

Intrinsically disordered proteins (IDP) and intrinsically disordered regions (IDR) are involved in many physiological mechanisms and pathologies. Although much effort has been dedicated towards understanding their structural and functional properties, IDPs and IDRs remain largely elusive. IDPs and IDRs contain a large proportion of polar, hydrophilic residues, compared to well-folded proteins and regions, which provide them the capability of populating a dynamic, rich ensemble of structures [1,2]. They do not fold spontaneously into a stable conformation in aqueous solvent because they lack a hydrophobic core or it is insufficiently large, but they are usually very susceptible to environmental factors (e.g., presence of ligands and interacting macromolecules, redox state, or pH of the surroundings) and posttranslational modifications [3]. Interestingly, they may fold into well-defined conformations when interacting with a biological partner or under appropriate conditions (the binding partner or concomitant processes, such as electron transfer or de/protonation, provide the additional physico-chemical context for establishing stabilizing interactions and triggering the disorder-to-order transition) or remain disordered (“fuzzy” complexes).

Disorder in proteins provides some unexpected advantages. First, disorder increases the structural plasticity and flexibility, allowing proteins to adopt multiple conformations within a complex conformational landscape depending on the environmental conditions, which is important for interacting with many biological partners [4,5] (in fact, IDPs or proteins containing IDRs represent important hubs in metabolic and signaling networks) and undergoing conformational changes required for cellular trafficking, internalization, and degradation [6,7]. Second, disorder makes some regions more susceptible to posttranslational modifications [8], which represents another protein regulation level conditioning the accessible conformations and the potential interactions with other biomolecules. Thirdly, disorder endows proteins with an additional regulation level based on allosteric control [9,10,11,12], which broadly consists in the regulation of the conformational landscape by ligand interaction (where ligand is any molecule interacting with a given protein: ion, small molecule, macromolecule.) [13,14].

Protein disorder has been maintained and exploited by evolution [15,16,17,18]. Therefore, it must be a key property that must be understood and accounted for when investigating structural and functional features in proteins. Some consequences derived from the presence of IDRs and the direct connection between order/disorder and function in proteins are not fully understood yet. Disordered regions in particular contribute to protein stability and function, sometimes with a counterbalancing effect [19,20], and should be considered as functional regions and not just flexible stretches [21,22].

The methyl-CpG binding protein 2, MeCP2, a multidomain protein containing a large proportion of intrinsic disorder and interacting with many biological partners, is a physiologically interesting and clinically relevant IDP. MeCP2 is involved in many key physiological processes associated with neuronal development, maturation, and plasticity [23,24], and MeCP2 deleterious mutations are associated with Rett syndrome, a neurodevelopmental disorder related to the autistic spectrum [25,26,27]. Although all domains must be relevant, because MeCP2 is a main transcriptional regulator and chromatin remodeling element, two domains emerge among the others: the methyl-binding domain (MBD) and the transcriptional repression domain (TRD). MBD is the domain responsible for the interaction with methylated-CpG-rich promoters [28,29,30]. Interestingly, MBD has a DNA binding site and contains one of the few well-structured regions in this protein, which is flanked by two completely disordered domains: N-terminal domain (NTD) and intervening domain (ID). Despite their lack of structure, both domains are critical for MeCP2 functions. In particular, mutations and posttranslational modifications in NTD affect and modulate MeCP2 functions [31,32]. In addition, the two identified MeCP2 isoforms differ in just a few residues located at the beginning of the NTD, but that small difference has important consequences in function, expression, and structural properties [33]. The ID not only provides a second DNA binding site, but it also considerably increases the DNA binding affinity of the MBD site.

It has been reported that both NTD and ID increase the intrinsic structural stability of MBD, as observed by thermal unfolding experiments: at pH 7, the unfolding temperature *T_m_* is 38.4 °C and 46.2 °C for MBD and NTD-MBD-ID, respectively, and the unfolding enthalpy Δ*H*_m_ is 38 kcal/mol and 46 kcal/mol for MBD and NTD-MBD-ID, respectively [34]. All other domains also have a minor stabilizing effect on MBD [35]. These unfolding experiments were undertaken by following the intrinsic fluorescence emission of the single tryptophan located at the MBD (W104), and consequently they accurately reflect the thermal stability of MBD. This stabilization effect is an intriguing outcome, considering that NTD and ID do not adopt well-defined structures by themselves. There are two main explanations for this phenomenon: (1) NTD and ID, although disordered or with high susceptibility to populate unfolded structures, are able to interact specifically with MBD or unspecifically (e.g., fuzzy interactions) (e.g., through long range polar/electrostatic interactions due to their large content in polar residues), thus increasing the intrinsic stability of MBD against unfolding; and (2) NTD and ID, being disordered and populating a multitude of different unfolded structures, may exert a steric hindrance effect on MBD, promote its compaction, and restrict its early expansion close to the unfolding temperature of the isolated MBD.

It might be speculated that the observed apparent stabilization effect exerted by NTD and ID could be some kind of artifact when determining the apparent stability of MBD due to: (1) using the intrinsic fluorescence of W104 as a local probe just reporting an unfolding process restricted to the vicinity of that residue; or (2) using temperature as a physico-chemical stress for altering the noncovalent interatomic interactions responsible for maintaining the folded MBD structure and, thus, triggering the protein unfolding process. In fact, a slightly destabilizing effect for NTD and ID on MBD was reported before [36], but minor differences in the protein constructs and/or experimental conditions could explain that disagreement. To rule out those possibilities, we have employed differential scanning calorimetry in order to use a thermal unfolding technique providing a global signal with contribution from the entire protein molecule, and we have also monitored the unfolding process by performing chemical denaturations using denaturant concentration as the physico-chemical stress triggering the protein unfolding process. Two MeCP2 constructions have been employed: the isolated MBD, and the MBD together with its two flanking domains, NTD-MBD-ID. The overall conclusion is that both completely disordered domains, NTD and ID, unequivocally stabilize the MBD against thermal and chemical denaturation. Therefore, disorder in proteins may be considered a pervasive feature that plays an important role in many the allosteric control of protein conformation, protein interactions, and protein regulation (modifications, trafficking, degradation, in/activation.). This may be even more important in multidomain IDPs with a complex conformational and multifunctional landscape [13,14].

## 2. Materials and Methods

### 2.1. Protein Expression and Purification

Protein variants (MBD and NTD-MBD-ID) were expressed and purified following identical procedures. Plasmids (pET30b) containing both constructions were transformed into BL21 (DE3) Star *E. coli* strain. Starting cultures were grown in 150 mL of LB/kanamycin (50 µg/mL) at 37 °C overnight. Then, 4 L of LB/kanamycin (25 µg/mL) were inoculated (1:100 dilution) and incubated under the same conditions until reaching an optical density (*λ* = 600 nm) of 0.6. Protein expression was induced by adding isopropyl 1-thio-β-d-galactopyranoside (IPTG) 1 mM at 18 °C overnight.

Cells were sonicated in ice and benzonase (Merck-Millipore, Madrid, Spain) was added (20 U/mL) to remove nucleic acids. Proteins were purified using metal affinity chromatography using a HiTrap TALON column (GE-Healthcare Life Sciences, Barcelona, Spain) with two washing steps: buffer sodium phosphate 50 mM, pH 7, NaCl 300 mM, and buffer sodium phosphate 50 mM, pH 7, NaCl 800 mM. Elution was performed applying an imidazole 10–150 mM elution gradient. Protein purity was evaluated by sodium dodecyl sulfate polyacrylamide gel electrophoresis.

The polyhistidine-tag was removed by processing with GST-tagged PreScission Protease in proteolytic cleavage buffer (Tris-HCl 50 mM, NaCl 150 mM, pH 7.5) at 4 °C for 4 h. Progress of the proteolytic processing was monitored by SDS-PAGE. The protein was further purified with a combination of two affinity chromatographic steps for removing the polyhistidine-tag (HiTrap TALON column) and the GST-tagged PreScission Protease (GST TALON column, from GE-Healthcare Life Sciences, Barcelona, Spain). Purity and homogeneity were evaluated by SDS-PAGE and size-exclusion chromatography. Storage buffer consisted of Tris 50 mM pH 7.0 and pooled samples were kept at −80 °C. The identity of all proteins was checked by mass spectrometry (4800plus MALDI-TOF/MS, from Applied Biosystems-Thermo Fisher scientific, Waltham, MA, USA). Potential DNA contamination was always estimated by UV absorption 260/280 ratio. Because a single tryptophan is located in MBD, an extinction coefficient of 11,460 M^−1^ cm^−1^ at 280 nm was employed for the two variants.

### 2.2. Double-Stranded DNA

HPLC-purified methylated and unmethylated 45-bp single-stranded DNA (ssDNA) oligomers, corresponding to the promoter IV of the mouse brain-derived neurotrophic factor (BDNF) gene [36,37], were purchased from Integrated DNA Technologies (Leuven, Belgium). Two complementary pairs of ssDNA were used for DNA binding assays:

forward unmethylated:
  5′-GCCATGCCCTGGAACGGAACTCTCCTAATAAAAG-ATGTATCATTT-3′;
reverse unmethylated:
  5′-AAATGATACATCTTTTATTAGGAGAGTTCCGTTCC-AGGGCATGGC-3′;
forward mCpG:
  5′-GCCATGCCCTGGAA(5-Me)CGGAACTCTCCTAATAAA-AGATGTATCATTT-3′;
reverse mCpG:
  5′-AAATGATACATCTTTTATTAGGAGAGTTC(5-Me)CGTT-CCAGGGCATGGC-3′.

The ssDNA oligonucleotides were dissolved at a concentration of 0.5 mM and the concentration was assessed by using the extinction coefficient provided by the manufacturer. Complementary ssDNA oligomers were mixed at an equimolar ratio and annealed to obtain 45-bp double-stranded DNA (dsDNA) using a Stratagene Mx3005P qPCR real-time thermal cycler (Agilent Technologies, Santa Clara, CA, USA). The thermal annealing profile consisted of: (1) equilibration at 25 °C for 30 s; (2) heating ramp up to 99 °C; (3) equilibration at 99 °C for 1 min; and (4) 3 h cooling process down to 25 °C at a rate of 1 °C/3 min.

### 2.3. Thermal Denaturation Assessment by Differential Scanning Calorimetry

Thermal stability of MeCP2 MBD and NTD-MBD-ID was assessed by temperature unfolding monitored by high-precision differential scanning calorimetry (DSC). The partial molar heat capacity of the protein in solution was measured as a function of temperature in an Auto-PEAQ-DSC (MicroCal, Malvern-Panalytical, Malvern, UK). Experiments were performed with a protein solution at a concentration of 20–40 μM in Tris 50 mM pH 7.0, and scanning from 15 to 95 °C at a scan rate of 60 °C/h.

The first approach in DSC experimental analysis consisted of applying a model-free data analysis for discriminating between different possibilities: two-state unfolding, non-two-state unfolding, and oligomer unfolding of the protein. From the thermogram (excess molar heat capacity of the protein as a function of the temperature, Δ*C*_P_ (*T*)), the calorimetric unfolding enthalpy, Δ*H*_cal_, the unfolding temperature, *T*_m_, and the maximal unfolding heat capacity, *C*_P,max_, were estimated, from which it was possible to calculate the van’t Hoff enthalpy, Δ*H*_vH_:

(1) $$\Delta H_{\mathrm{vH}}=\frac{4RT_{m}^{2}C_{P,\max}}{\Delta H_{\mathrm{cal}}}$$

From the ratio Δ*H*_vH_/Δ*H*_cal_ different possibilities may arise: (1) if Δ*H*_vH_/Δ*H*_cal_ = 1, the thermogram would reflect a single transition and the protein unfolds according to a two-state model (i.e., the protein contains a single energetic domain); (2) if Δ*H*_vH_/Δ*H*_cal_ < 1, the thermogram would reflect, at least, two (partially) overlapping transitions and the protein unfolds according to a non-two-state model (i.e., the protein contains, at least, two domains which unfold in an independent manner); and (3) if Δ*H*_vH_/Δ*H*_cal_ > 1, the thermogram would reflect an unfolding transition coupled to subunit dissociation (i.e., the protein is oligomeric and unfolds into monomers).

Once the appropriate unfolding model can be selected according to the previous van‘t Hoff test, the thermogram was analyzed by non-linear regression fitting analysis considering a set of *n* independent transitions, each characterized by a transition temperature *T*_m,i_, an unfolding enthalpy Δ*H*_i_ (*T*_m,i_), and an unfolding heat capacity Δ*C*_P,i_:

(2) $$\begin{array}{c} \langle \Delta C_{P}\left(T\right)\rangle =\overset{n}{\underset{i=1}{\sum }}\frac{K_{i}\left(T\right)}{{\left(1+K_{i}\left(T\right)\right)}^{2}}\frac{\Delta H_{i}{\left(T\right)}^{2}}{RT^{2}}\\ K_{i}\left(T\right)=\exp\left(-\Delta G_{i}\left(T\right)/RT\right)\\ \Delta G_{i}\left(T\right)=\Delta H_{i}\left(T_{m,i}\right)\left(1-\frac{T}{T_{m,i}}\right)+\Delta C_{P,i}\left(T-T_{m,i}-T\ln\frac{T}{T_{m,i}}\right) \end{array}$$

where *K*_i_, Δ*H*_i_, and Δ*G*_i_ are the equilibrium constant, the unfolding enthalpy, and the stabilization Gibbs energy for the protein conformational transition *i*, respectively, *R* is the ideal gas constant, and *T* is the absolute temperature.

### 2.4. Chemical Denaturation Assessment by Fluorescence Spectroscopy

Chemical stability of MeCP2 MBD and NTD-MBD-ID was assessed by fluorescence spectroscopy, using ultra-pure urea (urea crystalline pharma grade, PanReac, Barcelona, Spain) as chaotropic denaturant. The intrinsic fluorescence emission of the single tryptophan in MeCP2 (W104) was monitored inn a thermostated Cary Eclipse fluorescence spectrophotometer (Varian-Agilent, Santa Clara, CA, USA) using a protein concentration of 5 µM, in a 1 cm path-length quartz cuvette (Hellma Analytics, Müllheim, Germany). Experiments were performed in Tris 50 mM pH 7.0, at 20 °C, with temperature controlled by a Peltier unit. Fluorescence emission spectra were recorded from 300 to 500 nm, using an excitation wavelength of 290 nm and a bandwidth of 5 nm, at different urea concentrations ([*D*] = 0–7 M, with increments of 0.25 M). Samples were equilibrated overnight at room temperature before measurements. Guanidinium hydrochloride was avoided because it is a charged denaturant and would interfere with electrostatic interactions between protein charges and it is known to be a stabilizing molecule at low concentration [38,39]. Considering only the native and the unfolded states of the protein (two-state model) was sufficient for reproducing the denaturation curves, and inclusion of additional intermediate partially unfolded states was detrimental for the goodness of the fitting. Three unfolding-reporting signals were employed for quantitative analysis. The fluorescence emission intensity *I* at a single wavelength (in the case of MeCP2, 340 nm, i.e., *I*_340_), or the intensity ratio at two wavelengths (in the case of MeCP2, *I*_330_/*I*_350_) as a function of denaturant concentration, which, for an unfolding process involving independent transitions (corresponding to independently unfolding regions or domains), must be analyzed according to this set of equations:

(3) $$\begin{array}{c} \begin{array}{c} I\left(\left[D\right]\right)=\frac{1}{1+K\left(\left[D\right]\right)}I_{N}\left(\left[D\right]\right)+\frac{K\left(\left[D\right]\right)}{1+K\left(\left[D\right]\right)}I_{U}\left(\left[D\right]\right)\\ K\left(\left[D\right]\right)=\exp\left(-\Delta G\left(\left[D\right]\right)/RT\right) \end{array}\\ \begin{array}{c} \Delta G\left(\left[D\right]\right)=\Delta G_{w}\left(\left[D\right]\right)-m\left[D\right]\\ I_{i}\left(\left[D\right]\right)=A_{i}+B_{i}\left[D\right] \end{array} \end{array}$$

where *K*, Δ*G*, Δ*G*_w,_ and *m* are the equilibrium constant, the stabilization Gibbs energy in the presence and the absence of denaturant, and the susceptibility of Δ*G* to the denaturant concentration, for the conformational transition. The linear extrapolation model [40,41] has been assumed when accounting for the effect of denaturant concentration [*D*] on the stabilization energy, and the dependence of the intrinsic fluorescence intensity of each protein conformational state with the denaturant concentration was considered to be linear. The average emission energy of the spectrum <*E*> as a function of denaturant concentration, which must be analyzed in a similar way (Equation (3)), was also considered:

(4) $$\langle E\rangle =\frac{\sum_{i}I_{i}/\lambda_{i}}{\sum_{i}I_{i}}$$

where *I*_i_ is the fluorescence emission intensity in the spectrum at a certain wavelength *λ*_i_. Thus, <*E*> is the spectral average value of the inverse of the wavelength.

The advantage of using the fluorescence intensity is that is more quantitatively rigorous. However, the fluorescence intensity focuses on a certain region of the spectrum, while the average energy adds the possibility of taking into account changes along the entire spectrum for quantifying global spectral changes. Other unfolding observables, such as the wavelength for maximal intensity may lack proportionality with the advance of the unfolding process, may not change significantly upon unfolding, or may make the analysis more complex [42,43].

When needed, methylated and unmethylated dsDNA were added at 6 µM in order to test the ability of the protein to interact with dsDNA and evaluate the extent of the stabilization induced by DNA binding under the same conditions.

### 2.5. Dynamic Light Scattering (DLS)

Dynamic light scattering measurements were performed in a DynaPro Plate Reader III (Wyatt Technology, Santa Barbara, CA, USA) using a 384-multiwell plate (Aurora Microplates, Whitefish, MT, USA). Urea stock solution (8 M) was serially diluted to obtain concentrations ranging from 0 M to 7 M (steps of 1 M), before the addition of protein to a final concentration of 20 μM. All solutions were filtered using 0.2-μm membranes and protein stocks were centrifuged in microtubes for 2 min at maximum speed to prevent urea precipitation or protein aggregates from interfering with the DLS measurements. For each measurement, 10 acquisitions of 3 s were taken, and the apparent hydrodynamic radius was estimated from the experimental diffusion coefficient, obtained by the cumulant fit of the translational autocorrelation function, assuming a Rayleigh sphere model. Experiments were performed in Tris 50 mM pH 7.0, at 20 °C.

## 3. Results

### 3.1. The MBD Is Stabilized against Thermal Unfolding by Its Disordered Flanking Domains

**Figure 1:**
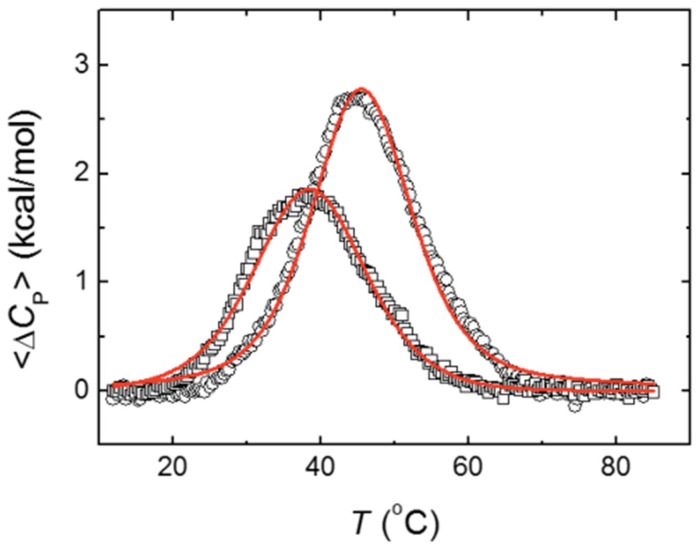
Differential scanning calorimetry thermograms (excess molar heat capacity of the protein as a function of temperature) for MBD (squares) and NTD-MBD-ID (circles). Experiments were done with a concentration of 40 μM, in Tris 50 mM, pH 7. Non-linear fittings are shown (red), according to a single transition model (for MBD) and a two independent transitions model (for NTD-MBD-ID).

The DSC thermograms showed a single apparent unfolding transition for MBD and NTD-MBD-ID (Figure 1). Experiments performed at 20 and 40 μM protein concentrations provided similar results (similar apparent *T*_m_ and unfolding enthalpy values), and therefore the native protein remains monomeric and the unfolding does not trigger oligomerization or aggregation.

The model-free van‘t Hoff analysis indicated that MBD unfolds as a single transition, whereas NTD-MBD-ID unfolds through two independent transitions, because the enthalpies ratio is close to 1 for MBD, but rather lower than 1 for NTD-MBD-ID (Table 1). The thermal unfolding monitored by fluorescence (using the single tryptophan W104 as an intrinsic probe) was previously analyzed according to a single transition (two-state model) and provided unfolding parameters in reasonable agreement with those reported now: transition temperature of 38.4 and 46.2 °C for MBD and NTD-MBD-ID, respectively, and unfolding enthalpy of 29 and 37 kcal/mol for MBD and NTD-MBD-ID, respectively. Thus, it seems that the presence of the two disordered flanking domains increased the stability of MBD and the unfolding process of the longer construct occurred with unfolding intermediates.

According to these results, the MBD unfolding was analyzed with a model considering a single transition and that for NTD-MBD-ID was analyzed with a model considering two independent transitions (Figure 1 and Table 2).

Although the unfolding of NTD-MBD-ID could be reasonably fitted with a single transition model, the residuals sum of squares (RSS) was smaller for the model with two transitions. More rigorously, the parametric F-test indicated that the model considering two transitions is statistically more appropriate (*F* = 50.40 > F_3213_ (α = 0.05) = 2.65). To observe other potential differences between MBD and NTD-MBD-ID, and to compare stabilities under different physico-chemical stresses, fluorescence chemical denaturations using urea as chaotropic denaturant were performed.

### 3.2. The MBD Is Stabilized against Chemical Unfolding by Its Disordered Flanking Domains

**Figure 2:**
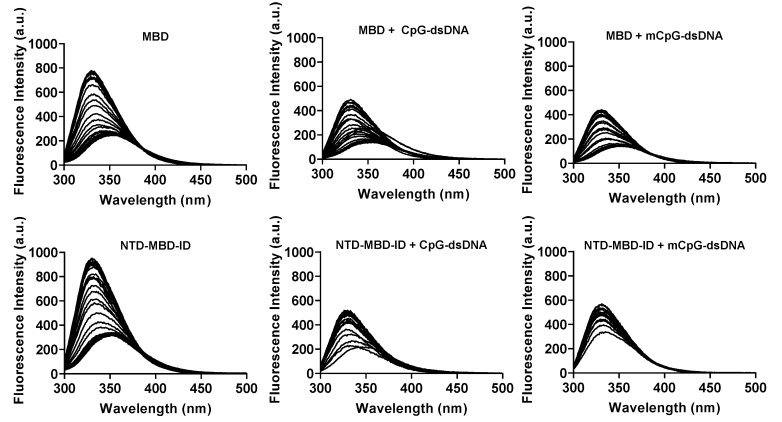
Raw fluorescence spectra for MBD (top) and NTD-MBD-ID (bottom) at different urea concentrations ([*D*] = 0–7 M) in the absence of dsDNA (**left**), in the presence of unmethylated CpG-dsDNA (**middle**), and methylated mCpG-dsDNA (**right**).

Because there is a single tryptophan residue (W104) in the whole MeCP2 sequence, and it is located in the MBD, measuring the intrinsic fluorescence of this residue is a good indicator of the folding state of MBD in both constructions. In the absence of denaturant, both protein constructs showed non-symmetrical, bell-shaped spectra, with a maximum around 340 nm indicating that W104 was not exposed to the solvent (Figure 2). As the concentration of denaturant increased, the intensity of emission was dramatically reduced (quenching) and red-shifted, with a maximum near 350 nm, as the fraction of unfolded protein increased, indicating that W104 was exposed to the solvent.

**Figure 3:**
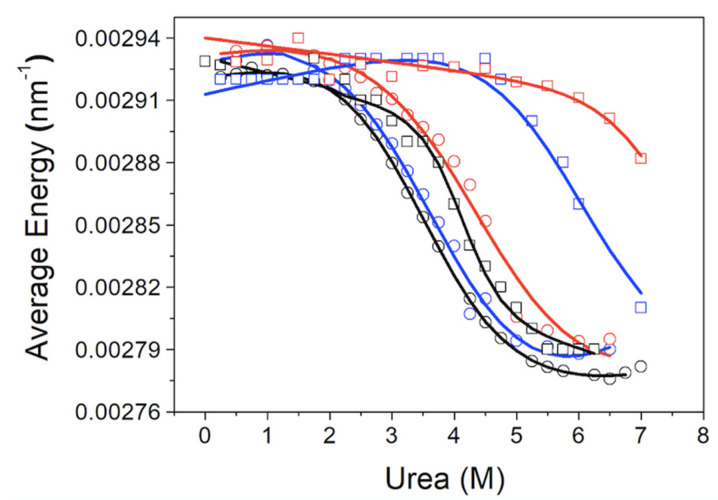
Chemical unfolding curves for MBD (circles) and NTD-MBD-ID (squares). The spectral average energy of the protein was represented as a function of the urea concentration: free protein (black), protein bound to unmethylated CpG-dsDNA (blue), and methylated mCpG-dsDNA (red).

Protein stabilization upon dsDNA was also determined by fluorescence chemical denaturation assays. When dsDNA was present, the emitted fluorescence intensity was considerably diminished due to light absorption by dsDNA at these wavelengths. Raw spectra were processed in order to calculate the spectral average energy at each experimental condition, and the unfolding traces were constructed (Figure 3).

The disordered flanking domains exerted a subtle stabilizing effect on MBD, as seen in the chemical unfolding curves monitoring the spectral average energy (Figure 3 and Table 3). The binding of dsDNA always produced stabilization of MBD against chemical denaturation (Figure 3 and Table 3).

The stabilizing effect was dramatically increased when the dsDNA was present. It must be born in mind that the presence of ID not only increases 400-fold the dsDNA binding affinity (from micromolar affinity to nanomolar affinity), but also provides an additional binding dsDNA site with micromolar affinity (the extent of the stabilization effect depends on the stoichiometry of the interaction and the binding affinity, among other factors) [34]. The interaction with mCpG-dsDNA was strikingly stabilizing for the protein. Indeed, even at high urea concentrations (i.e., [*D*] > 6 M), a large fraction of the protein (> 50%) seemed to remain folded. Unfolding traces could be satisfactorily analyzed employing an unfolding model with a single transition, and the unfolding parameters were estimated by non-linear least-squares regression analysis (Table 3).

**Figure 4:**
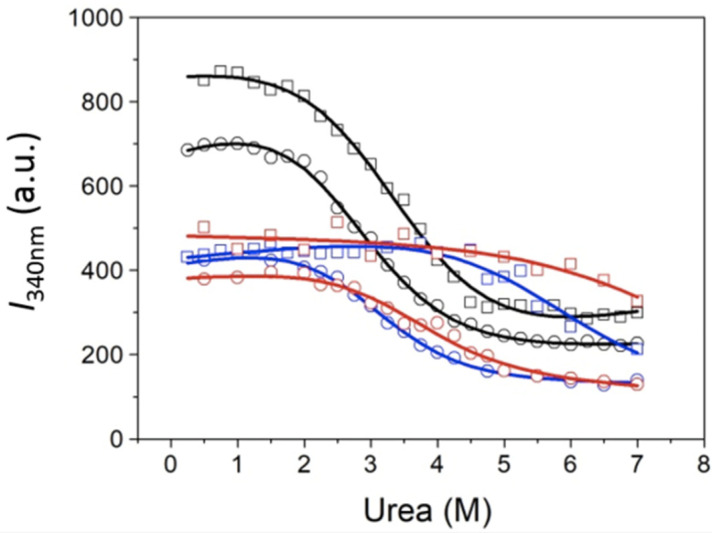
Chemical unfolding curves for MBD (circles) and NTD-MBD-ID (squares). The fluorescence intensity of the protein at 340nm was represented as a function of the urea concentration: free protein (black), protein bound to unmethylated CpG-dsDNA (blue), and methylated mCpG-dsDNA (red).

**Figure 5:**
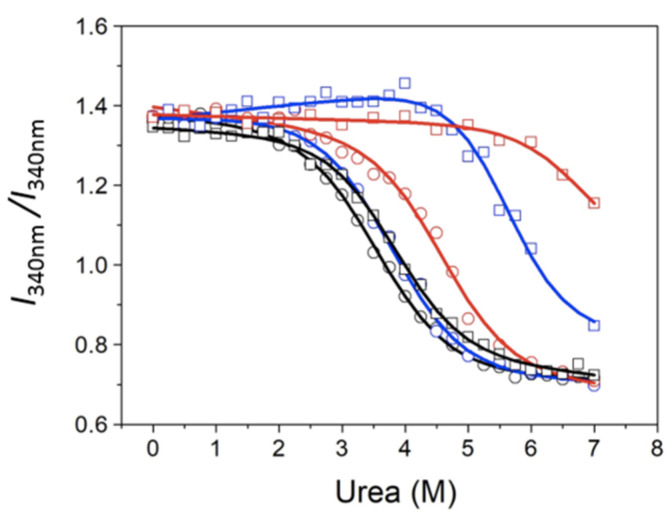
Chemical unfolding curves for MBD (circles) and NTD-MBD-ID (squares). The ratio of fluorescence intensities at 330 and 350 nm was represented as a function of the urea concentration: free protein (black), protein bound to unmethylated CpG-dsDNA (blue), and methylated mCpG-dsDNA (red).

For comparison, the spectral series shown in Figure 2 were also analyzed focusing on the fluorescence intensity at a single wavelength (340 nm), as shown in Figure 4 and Table 4, and the intensity ratio at two wavelengths (to reduce potential uncertainty and variability due to the protein concentration), as shown in Figure 5 and Table 5.

### 3.3. MBD Molecular Size Is Highly Susceptible to the Presence of the Flanking Domains

**Figure 6:**
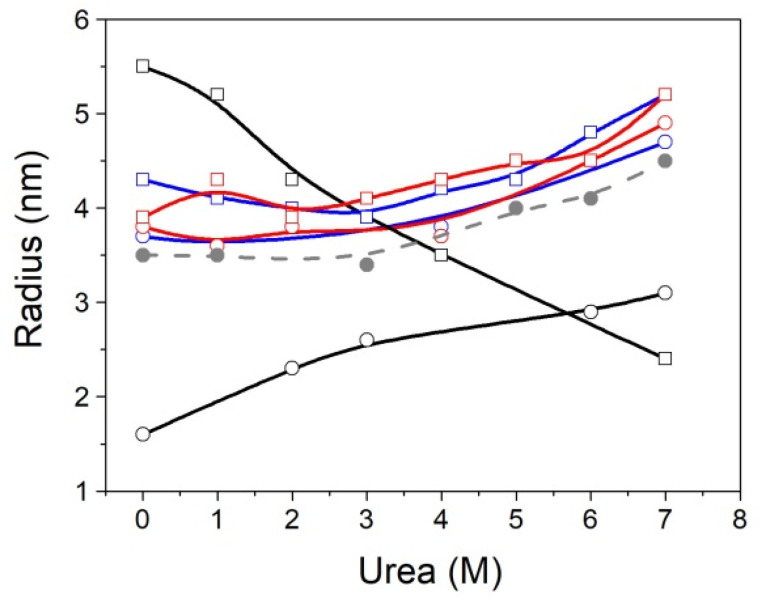
Apparent hydrodynamic radius of MBD and NTD-MBD-ID measured by DLS of MBD (empty circles) and NTD-MBD-ID (squares) in the absence of DNA (black), in the presence of unmethylated CpG-dsDNA (blue), and the presence of methylated mCpG-dsDNA (red). Control measurements of dsDNA are also shown (gray circles).

The apparent hydrodynamic radius of the two constructs, MBD and NTD-MBD-ID, was measured under different conditions: different urea concentrations and in the absence/presence of dsDNA (Figure 6 and Table 6). The size histograms showed average and standard deviation values that were modulated by urea concentration (Supplementary Figure S2). In addition, we could observe a similar effect from unmethylated and methylated dsDNA.

In the absence of urea, MBD showed a hydrodynamic radius close to that predicted from its molecular weight. As the concentration of urea increased, the apparent hydrodynamic radius of MBD increased, as expected for an unfolding process, but the apparent hydrodynamic radius of NTD-MBD-ID decreased. This unexpected result may be related to the large proportion of disorder, the large proportion of charged residues, and the increase in dielectric constant of the solvent when urea concentration increases, as discussed later. The interpretation of the low susceptibility and small increase of the apparent hydrodynamic radius of the two constructs to the concentration of urea in the presence of dsDNA is difficult, because it must be a combination of several effects: initial stabilization of protein by dsDNA interaction is weakened because of the reduction in the dielectric constant of the solvent (weakened polar/electrostatic interactions between protein and dsDNA) and the preferential interaction of urea with the protein residues displacing water molecules, leading to dsDNA dissociation and protein destabilization and unfolding.

## 4. Discussion

The impact of disordered regions on the stability and functional features of well-folded regions in proteins remains as an important and elusive matter intimately connected with protein function regulation and allosteric control. There are some cases where this issue gets especially important. For example, the stabilization induced by intrinsically disordered regions in the HIV-1 Rev protein has been reported [21]. In another example, stability changes have been described for nucleoplasmin depending on the length of the disordered tail in each subunit of the pentameric protein: a fifty-residue C-terminal deletion mutant showed lower thermal stability, whereas an eighty C-terminal deletion mutant showed higher thermal stability than the full-length protein [22]. In the case of MeCP2, it has been previously outlined that the two flanking domains of MBD in MeCP2 (NTD and ID) increase MBD stability and dsDNA binding affinity. This is not a simple issue, because those two domains are fully disordered and MBD is also 40% disordered, approximately. The comparison of the circular dichroism spectra for both constructions reflected a lower level of structural order for NTD-MBD-ID (Supplementary Figure S1). The stabilization effect was observed through fluorescence thermal denaturations, and two reasonable objections could be pointed out: (1) the lack of a signal reporting global effects in the protein, because fluorescence intensity only reports the local effect in the surroundings of the single tryptophan in MBD in the thermal denaturations, and (2) the thermal stress driving the unfolding process that could result in an artifactual observation, whereas other unfolding processes (e.g., chemical or pressure denaturation) might provide a somewhat different outcome. In order to rule out these possibilities, we studied the unfolding of MBD and NTD-MBD-ID by differential scanning calorimetry and by chemical denaturation.

Both constructions, MBD and NTD-MBD-ID, showed a single apparent transition in their DSC thermogram. Applying the van ‘t Hoff (two-state) test, it could be observed that, although MBD seemed to unfold through a single transition, the unfolding of NTD-MBD-ID involved at least two intermediate unfolding states that can be significantly populated at moderate temperatures. Thus, the conformational landscape of NTD-MBD-ID is more complex than expected, and its unfolding cooperativity is lower than that of MBD. The two unfolding transitions observed in NTD-MBD-ID might correspond to two independent transitions within MBD (the presence of the flanking domains decouples two regions and lowers the unfolding cooperativity of MBD) or might reflect a transition in NTD or ID corresponding to a region that adopts a folded conformation when accompanying the MBD and undergoes unfolding.

**Figure 7:**
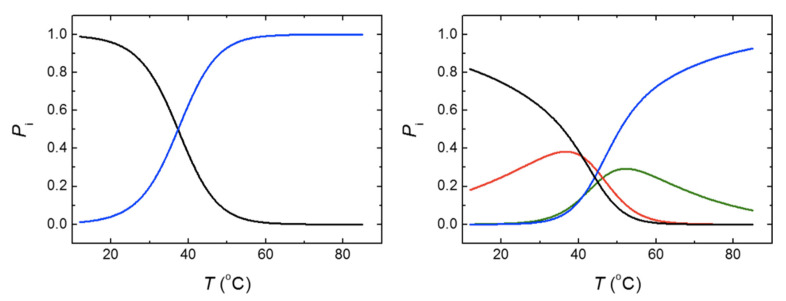
Molar fraction or population (*P*_i_) of the different conformational states: native state (black), first intermediate state (red), second intermediate state (green), and fully unfolded state (blue), for MBD (left) and for NTD-MBD-ID (right). Populations were calculated with the parameters shown in Table 2.

The stabilization effect exerted by NTD and ID was evident from the comparison of the apparent transition temperatures for both constructs determined by DSC, namely 37.4 °C for MBD and 45 °C for NTD-MBD-ID, which are fairly similar to those determined by fluorescence thermal denaturations (38.4 °C for MBD and 46.2 °C for NTD-MBD-ID). However, a striking phenomenon can be noticed with a further analysis. From the unfolding parameters reported in Table 2, the molar fractions of the relevant conformational states for MBD and NTD-MBD-ID at any temperature can be outlined (Figure 7). It can be observed that at 20 °C, 96% of MBD remains in its native conformation, and the temperature for the unfolded state to be 50% populated was 37.4 °C (the transition temperature). From that, a stabilization Gibbs energy at 20 °C of 1.9 kcal/mol could be estimated, in fair agreement with the 1.4 kcal/mol estimated by thermal fluorescence denaturations. However, although the completely unfolded state of NTD-MBD-ID reaches a 50% population at 50.7 °C, the native state is populated only 75% at 20 °C with a corresponding stabilization Gibbs energy of just 0.6 kcal/mol, because of the coexistence of partially unfolded states. Therefore, it is obvious that NTD-MBD-ID requires higher temperatures for complete unfolding compared to MBD, but at low temperature the relevant stability (the stabilization energy gap connecting the native state and the first partially unfolded state) [44] is lower than that of MBD (0.6 kcal/mol for NTD-MBD-ID and 1.9 kcal/mol for MBD), and the integrity of the NTD-MBD-ID native state is compromised to a larger extent at low temperatures. For example, at 37.4 °C (a temperature at which the stabilization Gibbs energy for MBD is zero and both native state and unfolded state are 50% populated), the stabilization Gibbs energy of NTD-MBD-ID is –0.7 kcal/mol and the native state is only 46% populated.

The lack of correlation between the apparent *T*_m_’s and the stabilization Gibbs energy at low temperature is an indication of the caveats associated with using only *T*_m_’s for assessing structural stability. A rigorous stability assessment must involve the determination of stabilization Gibbs energies at relevant conditions. This problem parallels with the often-observed lack of correlation between the half-unfolding denaturant concentration [*D*]_1/2_ and the stabilization Gibbs energy Δ*G*_w_, as discussed below.

Temperature and chemical denaturation modulate and alter intramolecular noncovalent interactions (e.g., hydrogen bonds, electrostatic interactions and salt bridges, hydrophobic interactions) by different mechanisms. Temperature alters the thermal motion, lowers the barrier to overcome the stabilizing interatomic energies, and reduces the dielectric constant, with an overall destabilizing effect [45]. Urea increases the dielectric constant (higher than water) [46], diminishing the strength of polar/charge interactions, and interacts specifically with the protein backbone and displaces water molecules by preferential interaction lowering the hydration capability of water, destabilizing the native state, and shifting the conformational equilibrium towards (partially and completely) unfolded states, with an overall destabilizing effect. The different effect of temperature on the dielectric constant (and therefore, on the strength of electrostatic interactions) is relevant, since IDPs such as MeCP2 contain a considerable percentage of polar/charged residues, and specific and unspecific electrostatic interactions may be established intramolecularly. Thus, electrostatic interactions, either specific direct interactions or long-range unspecific interactions, surely play an important role mediating the reciprocal effect between MBD and both flanking domains NTD and ID. An interesting observation arises from the fact that the stabilization effect is considerably diminished at pH 7 when the ionic strength is increased: at NaCl 150 mM, the unfolding temperature *T*_m_ is 46.4 °C and 49.8 °C for MBD and NTD-MBD-ID, respectively, and the unfolding enthalpy Δ*H*_m_ is 32 kcal/mol and 38 kcal/mol for MBD and NTD-MBD-ID, respectively. IDPs and IDRs are rich in polar/charged residues, and the ionic screening on electrostatic charges will affect both the specific and the unspecific mechanisms of interdependence between folded and unstructured domains.

The chemical denaturation of MBD and NTD-MBD-ID followed by fluorescence seem to occur as a single transition. From the experimental data, a clear stabilization effect of NTD and ID can be observed from the apparent half-unfolding denaturant concentration: [*D*]_1/2_ is 3.30 M for MBD and 3.95 for NTD-MBD-ID. However, when calculating the stabilization Gibbs energy Δ*G*_w_, the difference is not as clear, and the changes observed in [*D*]_1/2_ be obscured in Δ*G*_w_ because of the *m* values. A similar phenomenon occurs when calculating stabilization Gibbs energies at low temperature from the T_m_, in which the unfolding heat capacity and enthalpy values are critical for a correct extrapolation. Therefore, estimating stabilities just focusing on *T*_m_ or [*D*]_1/2_ is risky and limited; stabilization energies at a reference experimental condition (e.g., 20 °C and absence of denaturant) must be determined when assessing structural stability.

As previously reported, focusing on different observable signals may result in considerable differences in estimated stability parameters from spectroscopic unfolding curves [42]. In the case of MeCP2, it seems that the analysis based on the fluorescence intensity at 340 nm underestimates the stabilization energies for NTD-MBD-ID in the presence of dsDNA, but the corresponding unfolding traces are ill-defined in the post-transition region because of the high stability. Moreover, the analysis based on the fluorescence intensity ratio seems to overestimate stabilization energies for most of the cases. In this respect, it must be born in mind that the intensity ratio might lack proportionality with the advance of the unfolding process [47,48]. Although the absolute values of stabilization energies do not completely agree, the differences in stabilization energies between protein constructs are rather independent of the signal employed; in particular, the presence of NTD and ID increases the stability of MBD by 0.4 kcal/mol. Thus, even considering the present limitations, the reported data confirm the stabilizing effect of the disordered domains, NTD and ID, on the stability of MBD.

The previously reported stabilization Gibbs energies against thermal denaturation [34] are similar to those determined from chemical denaturation, but they are not in full agreement. At 20 °C, MBD showed a stabilization energy of 1.4 kcal/mol determined from fluorescence thermal denaturations, while it showed a stabilization energy of 2.5 kcal/mol determined by chemical denaturation. Moreover, NTD-MBD-ID showed a stabilization energy of 2.5 kcal/mol determined from fluorescence thermal denaturations, while it showed a stabilization energy of 2.8 determined by chemical denaturation. Of course, those quantities do not necessarily must be in agreement, because, although the initial state in both unfolding processes is the same (the native state), the final state may be different: the unfolded state after thermal denaturation may be structurally different from the unfolded state after chemical denaturation [49,50]. The same occurs with the unfolding process itself, which may proceed through different routes and involving different intermediate states depending on whether temperature or denaturant concentration drives the unfolding process.

**Figure 8:**
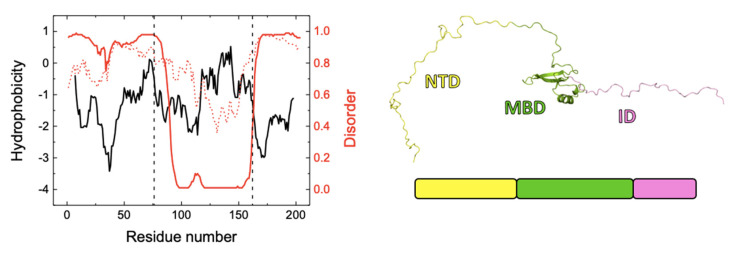
(Left) Hydrophobicity (Kyte & Doolittle scale) plot for NTD-MBD-ID (black) calculated using Expasy ProtScale tool (https://web.expasy.org/protscale/, accessed on 1 July 2021), and disorder prediction plots according to DISOPRED3 (red, continuous line) and IUPRED2 (red, dotted line). The separation between the three domains, NTD, MBD, and ID, are indicated by the vertical dashed lines. (Right) One of the possible conformational states of NTD-MBD-ID, showing the two disordered domains, NTD (yellow) and ID (pink), flanking the folded domain (MBD).

Regarding the DLS data, MBD showed a hydrodynamic radius similar to that predicted by size-scaling based on molecular weight, but NTD-MBD-ID showed a much larger hydrodynamic radius than the predicted one [51,52]. If MBD were completely folded or unfolded, the hydrodynamic radius would be expected to be around 1.9 nm or 2.9 nm, respectively, and the observed radius is 1.6 nm; therefore, MBD must contain a considerable percentage of folded structure, as expected. However, NTD-MBD-ID showed an abnormally large radius (5.5 nm), much larger than that predicted even if fully unfolded. If NTD-MBD-ID were completely folded or unfolded, the hydrodynamic radius would be expected to be around 2.5 nm or 4.6 nm. Therefore, assuming the agreement is reasonable (considering the experimental uncertainties and important approximations applied when estimating the apparent hydrodynamic radius by DLS, as well as the uncertainties associated with estimating the size from the protein molecular weight), NTD-MBD-ID must contain a very large proportion of disorder. It is interesting to point out that the completely unfolded NTD is expected to have a hydrodynamic radius of around 2.6 nm, and the completely unfolded ID is expected to have a hydrodynamic radius of around 1.8 nm. Figure 8 shows the hydrophobicity profile of NTD-MBD-ID, which shows two hydrophobic regions, at the end of NTD and the second half of MBD. Disorder predictions (by DISOPRED3 and IUPRED2 algorithms [53,54]) reveal that structural order is confined to most of MBD, and NTD and ID may remain mostly unstructured.

The influence of NTD and ID on MBD may be twofold: (1) unspecific, through steric constraining and long-range electrostatic interactions, and (2) specific, through direct interaction with certain regions in the MBD. Both phenomena would result in spatial restriction of the MBD and additional stabilization, hindering its ability to unfold at moderate temperatures and denaturant concentrations. The interdependence between the different domains in MeCP2 was observed before. Acrylamide collisional quenching, which reports the solvent accessibility of the single tryptophan in MeCP2 (located in MBD), revealed the structural coupling between MBD and all other domains [36]. The structure of the MBD was influenced other domains leading to the shielding of W104 from solvent exposure, with all domains contributing, especially NTD, ID, and TRD.

If the influence of the disordered domains on MBD were mostly due to electrostatic interactions, the ionic strength would strongly modulate the structural stability of MBD. It was reported that increasing the intrinsic ionic strength (NaCl 150 mM) decreases the stabilization effect of the disordered flanking domains (Δ*T*_m_ = 7.8 °C with zero ionic strength compared to Δ*T*_m_ = 3.4 °C with NaCl 150 mM). However, there is still a substantial stabilization effect even though electrostatic interactions are screened. This suggests the steric/conformational effects are important for the stabilization effect on MBD.

Regarding the experiments with dsDNA, the same phenomena previously observed by fluorescence thermal denaturations were also observed by chemical denaturations: the stabilization effect induced by dsDNA, the larger stabilization effect of dsDNA on NTD-MBD-ID compared to that on MBD, and the larger stabilization exerted by methylated mCpG-dsDNA compared to unmethylated CpG-dsDNA [34]. Still, there is no clue about the underlying mechanism by which such a moderate difference in the in vitro affinity of MeCP2 for dsDNA between the two methylation states (less than five-fold difference in affinity [34]) is further translated to a considerable difference in stabilization upon binding. This hierarchy we have seen in the unfolding experiments is more coherent with the in vivo situation, where MeCP2 acts as a consistent reader of methylation in chromatin.

## 5. Conclusions

In multidomain proteins, there must be a reciprocal influence of the different constitutive domains, an effect that may be qualitatively and quantitatively different in IDPs because of the larger dynamic sampling of structurally diverse conformational states. Considerable flexibility and a highly dynamic conformational landscape, large susceptibility to environmental conditions, and potential interactions with partners might cause this interdependence to be conditional and dependent on many intervening factors, as well as provide an additional regulation level for protein conformation and function (i.e., allosteric control). MeCP2 is a multidomain IDP in which MBD, the main domain responsible for DNA recognition, is substantially influenced by its flanking disordered domains. Not only does NTD-MBD-ID differ functionally from MBD, but NTD-MBD-ID also shows differential structural features compared to MBD: (1) conformational landscape with higher complexity, where partially unfolded states may be (functionally) relevant; (2) lower unfolding cooperativity due to the coexistence of partially unfolded intermediate states; and (3) lower relevant stability than MBD but higher overall stability at moderate temperature. In addition, MBD mutations associated with Rett syndrome have different impacts depending on the molecular context, the isolated MBD or the NTD-MBD-ID construct [55]. The application of other experimental techniques (e.g., nuclear magnetic resonance, small-angle X-ray scattering) and computational studies applying molecular dynamic simulations with MBD and NTD-MBD-ID remain very challenging considering the disordered nature of the protein, which might reveal additional effects on MBD from NTD and ID. Moreover, it is very likely that the complexity of the behavior of MeCP2 will be higher for the full-length protein containing the six structural domains.

## Figures and Tables

**Table 1:** Parameters associated with the van ‘t Hoff test (or calorimetric two-state test).

Protein Construction	*T*_m_ (°C)	Δ*H*_cal_ (kcal/mol)	*C*_Pmax_ (kcal/K·mol)	Δ*H*_vH_ (kcal/mol)	Ratio
MBD	37.8	36	1.85	39	1.08
NTD-MBD-ID	44.8	52	2.73	42	0.81

**Table 2:** Thermal unfolding parameters for MBD and NTD-MBD-ID at pH 7.

Protein Construction	*T*_m1_ (°C)	Δ*H*_1_ (kcal/mol)	*T*_m2_ (°C)	Δ*H*_2_ (kcal/mol)	Sqrt(RSS) (kcal/mol)
MBD	37.4 ± 0.2	38 ± 1	--	--	1.0603
NTD-MBD-ID	45.0 ± 0.2	47 ± 1	--	--	2.0606
	41.7 ± 0.2	11 ± 1	45.0 ± 0.2	46 ± 1	1.5540

Sqrt(RSS): square root of the residual sum of squares.

**Table 3:** Thermal unfolding parameters for MBD and NTD-MBD-ID at pH 7, estimated by by analyzing the denaturant dependence of the spectral average energy.

Protein Construction	DNA	Δ*G*_w_ (kcal/mol)	*m* (kcal/mol·M)	[*D*]_1/2_ (M)
MBD	--	2.5	0.77	3.3
	CpG-dsDNA	2.7	0.68	3.9
	mCpG-dsDNA	3.2	0.67	4.8
NTD-MBD-ID	--	2.8	0.70	4.0
	CpG-dsDNA	4.2	0.72	5.9
	mCpG-dsDNA	7.6	0.97	7.8

[*D*]_1/2_ is the half-unfolding denaturant concentration. The stabilization Gibbs energy in the absence of denaturant is related to [*D*]_1/2_: Δ*G*_w_ = *m* [*D*]_1/2_.

**Table 4:** Thermal unfolding parameters for MBD and NTD-MBD-ID at pH 7, estimated by analyzing the denaturant dependence of the fluorescence intensity at 340 nm.

Protein Construction	DNA	Δ*G_w_* (kcal/mol)	*m* (kcal/mol·M)	[*D*]_1/2_ (M)
MBD	--	2.3	0.84	2.7
	CpG-dsDNA	2.7	0.92	2.9
	mCpG-dsDNA	2.9	0.83	3.5
NTD-MBD-ID	--	2.7	0.78	3.4
	CpG-dsDNA	3.6	0.64	5.6
	mCpG-dsDNA	3.8	0.49	7.7

[*D*]_1/2_ is the half-unfolding denaturant concentration. The stabilization Gibbs energy in the absence of denaturant is related to [*D*]_1/2_: Δ*G*_w_ = *m* [*D*]_1/2_.

**Table 5:** Thermal unfolding parameters for MBD and NTD-MBD-ID at pH 7, estimated by analyzing the denaturant dependence of the fluorescence intensity ratio *I*_330_/*I*_350_.

Protein Construction	DNA	Δ*G*_w_ (kcal/mol)	*m* (kcal/mol·M)	[*D*]_1/2_ (M)
MBD	--	3.6	1.02	3.5
	CpG-dsDNA	3.9	1.04	3.7
	mCpG-dsDNA	4.8	1.06	4.6
NTD-MBD-ID	--	4.1	1.09	3.8
	CpG-dsDNA	6.9	1.24	5.6
	mCpG-dsDNA	7.7	1.10	7.0

[*D*]_1/2_ is the half-unfolding denaturant concentration. The stabilization Gibbs energy in the absence of denaturant is related to [*D*]_1/2_: Δ*G*_w_ = *m* [*D*]_1/2_.

**Table 6:** Hydrodynamic radius of the two protein constructs in complex with dsDNA estimated by dynamic light scattering.

Protein Construction	DNA	Radius (nm)
MBD	--	1.6
	CpG-dsDNA	3.7
	mCpG-dsDNA	3.8
NTD-MBD-ID	--	5.5
	CpG-dsDNA	4.3
	mCpG-dsDNA	3.9
--	CpG-dsDNA	3.5

## Data Availability

The data presented in this study are available upon reasonable request from the corresponding author.

## Supplementary Materials

The following are available online at https://www.mdpi.com/article/10.3390/biom11081216/s1, Figure S1: Circular dichroism, Figure S2: DLS size histograms.
