# Supplementary material for: Stabilization Effect of Intrinsically Disordered Regions on Multidomain Proteins: The Case of the Methyl-CpG Protein 2, MeCP2
Source: Biomolecules. 2021 Aug 16;11(8):1216. doi: 10.3390/biom11081216 (PMC8391517; doi:10.3390/biom11081216)
Supplement: Supplementary file 1 [file biomolecules-11-01216-s001.zip › biomolecules-1315098-supplementary.pdf]

biomolecules

MDPI

Article

Stabilization effect of intrinsically disordered regions on multidomain proteins: The case of the methyl-CpG protein 2, MeCP2

David Ortega-Alarcon [1], Rafael Claveria-Gimeno [1,2,3], Sonia Vega [1], Olga C. Jorge-Torres [4], Manel Esteller [4,5,6,7], Olga Abian [1,2,3,8,9*] and Adrian Velazquez-Campoy [1,3,8,9,10*]

[1] Institute of Biocomputation and Physics of Complex Systems (BIFI), Joint Units IQFR-CSIC-BIFI, and GBsC-CSIC-BIFI, Universidad de Zaragoza, Zaragoza, 50018, Spain; dortega@bifi.es (D.O.-A.); svega@bifi.es (S.V.); rafacg@certest.es
[2] Instituto Aragonés de Ciencias de la Salud (IACS), 50009, Zaragoza, Spain
[3] Instituto de Investigación Sanitaria de Aragón (IIS Aragón), 50009, Zaragoza, Spain
[4] Josep Carreras Leukaemia Research Institute (IJC), 08916, Badalona, Spain; ojorge@carrerasresearch.org (O.C.J.-T.); mesteller@carrerasresearch.org (M.E.)
[5] Centro de Investigacion Biomedica en Red Cancer (CIBERONC), 28029, Madrid, Spain
[6] Institucio Catalana de Recerca i Estudis Avançats (ICREA), 08010, Barcelona, Spain
[7] Physiological Sciences Department, School of Medicine and Health Sciences, University of Barcelona (UB), l'Hospitalet de Llobregat, 08907, Barcelona, Spain
[8] Centro de Investigación Biomédica en Red en el Área Temática de Enfermedades Hepáticas y Digestivas (CIBERehd), 28029, Madrid, Spain
[9] Departamento de Bioquímica y Biología Molecular y Celular, Universidad de Zaragoza, 50009, Zaragoza, Spain
[10] Fundación ARAID, Gobierno de Aragón, 50009, Zaragoza, Spain
* Correspondence: oabifra@unizar.es (O.A.); adrianvc@unizar.es (A.V.-C.); Tel.: +34-876-555417 (O.A.); +34-976-762996 (A.V.-C.)
† Current address: Certest Biotec S.L., 50840, Zaragoza, Spain

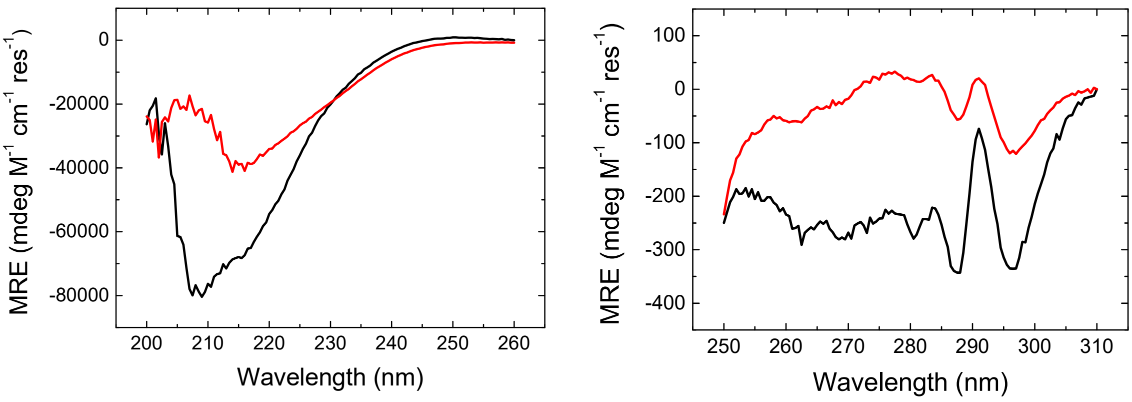

Figure S1. Circular dichroism. Far-UV (left) and near-UV (right) circular dichroism spectra of MBD (black) and NTD-MBD-ID (red).

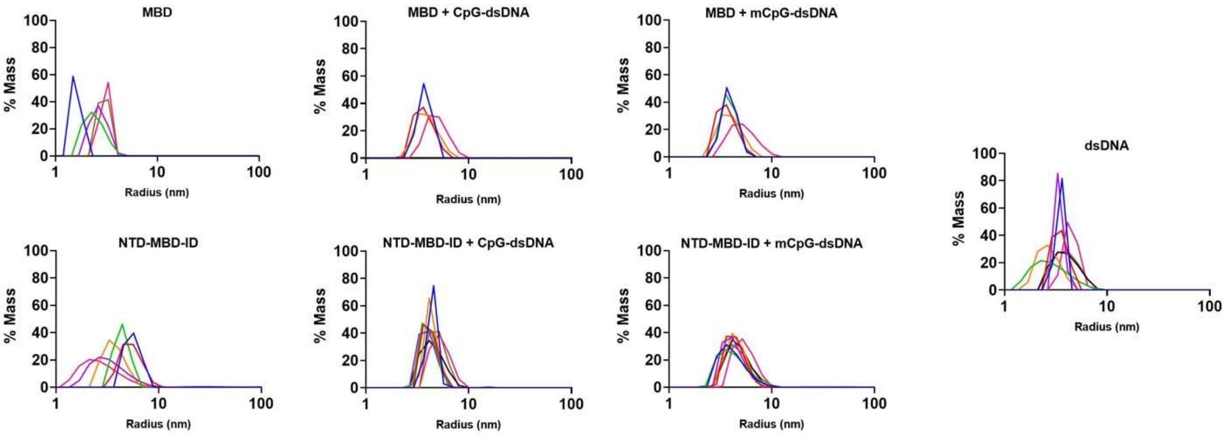

Figure S2. DLS size histograms. Size distribution histograms for MBD and NTD-MBD-ID in the absence of dsDNA and the presence of unmethylated and methylated dsDNA, at different urea concentrations: 0 M (blue), 1 M (red), 2 M (green), 3 M (violet), 4 M (orange), 5 M (black), 6 M (brown), and 7 M (pink).
